# Screening for atrial fibrillation with or without general practice involvement: a controlled study

**DOI:** 10.1186/s12875-025-02878-y

**Published:** 2025-05-26

**Authors:** Rakesh N. Modi, Efthalia Massou, Peter H. Charlton, Andrew Dymond, Kate Williams, James Brimicombe, Ben Freedman, Simon J. Griffin, F. D. Richard Hobbs, Gregory Y. H. Lip, Richard J. McManus, Jonathan Mant

**Affiliations:** 1 Department of Public Health and Primary Care, Primary Care Unit, University of Cambridge, Strangeways Research Laboratory, 2 Worts’ Causeway, Cambridge, CB1 8RN UK; 2 Heart Research Institute, University of Sydney, Room 3114, Level 3 East, D17 - Charles Perkins Centre, Sydney, NSW 2006 Australia; 3 MRC Epidemiology Unit, School of Clinical Medicine, University of Cambridge, Cambridge Biomedical Campus, Cambridge, CB2 0SL UK; 4 Nuffield Department of Primary Care Health Sciences, University of Oxford, Radcliffe Observatory Quarter, Woodstock Road, Oxford, OX2 6GG UK; 5 Liverpool Centre for Cardiovascular Science at University of Liverpool, Liverpool John Moores University and Liverpool Heart & Chest Hospital, Liverpool, UK; 6 Department of Clinical Medicine, Aalborg University, Aalborg, Denmark; 7 Brighton and Sussex Medical School, University of Brighton and University of Sussex, Brighton, UK

**Keywords:** Screening, Atrial fibrillation, Feasibility study, Primary care, Remote deliver

## Abstract

**Background:**

There has been a drive to increase atrial fibrillation (AF) detection in general practice. However, one-off, opportunistic testing can miss paroxysmal AF and requires significant resource. Paroxysmal AF can be detected through screening that involves repeated ECGs over a period of time, although it is unclear whether screening would need to be led by general practice, and how much support participants require. We aimed to investigate whether AF screening can be delivered remotely by a centralised administration instead of general practice, and to determine the level of support required.

**Methods:**

We undertook a controlled comparator study with secondary randomisation in three English general practices. We invited people aged ≥ 70 years to use a hand-held ECG device four times daily for three weeks. Participants were allocated to practice-led or administrator-led screening, with administrator-led support randomised to three different levels. We compared quantity and quality of ECGs obtained in each arm. The primary outcome was proportion of screened participants who recorded ≥ 56 adequate-quality ECGs (2/3 of possible ECGs).

**Results:**

Of 288 screened participants, 59 participants received practice-led screening with a telephone consultation to explain the device. The remainder received administrator-led screening: 81 were automatically given a consultation; 74 were offered a consultation, and 74 were not offered a consultation. Most screened participants (280/288, 97.2%) recorded ≥ 56 adequate-quality ECGs. This proportion did not vary significantly between practice-led and administrator-led screening (100.0% vs. 98.8%), or between support levels (94.6% to 98.8%). Practice-led screening led to slightly more adequate-quality ECGs (mean: 83.9 vs 78.3, *p* < 0.001).

**Conclusions:**

AF screening can be successfully delivered remotely, outside general practice, with minimal support.

**Supplementary Information:**

The online version contains supplementary material available at 10.1186/s12875-025-02878-y.

## Introduction

The UK National Health Service (NHS) long term plan emphasises detecting more cases of atrial fibrillation (AF) to reduce the burden of stroke [1]. Although there is currently no national AF screening programme in place, [2] some screening via opportunistic pulse checks or ECGs using hand-held ECG devices has been encouraged [3, 4]. The workload has largely fallen on general practice [3, 5–8]. However, general practice is already under pressure and has a shortage of appointments [9]. Furthermore, one-off screening in clinic will miss cases of paroxysmal AF that are also associated with stroke. [10, 11] An alternative approach is to undertake intermittent screening at home with minimal involvement of general practice.

A centralised administration of non-clinical staff outside of general practice is an alternative location for this work. Some screening programmes are already remote and delivered by centralised administrators such as the NHS Bowel Cancer Screening Programme [12]. The pandemic has catalysed more such possibilities [13] These include remote blood pressure monitoring, [13, 14] cervical screening self-sampling, [15] and sexual infection screening [16, 17]. It is therefore conceivable that screening for AF could be delivered remotely by centralised administrators rather than general practice. However, this, and the amount of patient support needed, has not been tested.

The Screening for Atrial Fibrillation with ECG to Reduce stroke (SAFER) randomised controlled trial seeks to determine whether systematic screening for paroxysmal AF with a hand-held single-lead ECG device reduces stroke [18]. Screening for paroxysmal AF has been shown to identify more AF than usual care, but whether this has an impact on stroke is unclear. [19, 20] The programme was originally conceived as a general practice-based intervention whereby practice nurses would train patients in face-to-face appointments on intermittently recording their own ECGs at home. We demonstrated the feasibility of this approach in pre-pandemic feasibility studies [19]. As a response to the COVID-19 pandemic, we carried out an additional feasibility study that demonstrated that patients could use a hand-held ECG successfully at home without the need for a face-to-face appointment [21]. Here we report data from our additional feasibility study to investigate whether AF screening could be delivered outside of primary care, and with minimal support.

We compared delivery of AF screening by general practice and by centralised administrators, with varying levels of support. This study of how best to deliver such a programme contributes evidence towards the UK National Screening Committee’s ‘Implementation criteria’ when assessing whether a screening programme should be recommended [22].

Method.

### Aim

To explore the impact of different models of delivery of at-home screening for AF on the quality and quantity of ECGs self-recorded by participants.

### Design

A controlled and part-randomised interventional study with four arms.

### Setting

This study took place from October to December 2020, preceding the internal pilot of the SAFER trial (ISRCTN72104369, registered 11 th March 2020) [18]. This occurred during COVID-19 restrictions when remote consulting was recommended [23]. We recruited participants from three East of England general practices identified by the National Institute for Health and Care Research (NIHR) Clinical Research Network (CRN) as experienced in delivering studies.

### Participants

Eligible participants were identified by searches of the general practice electronic records (people aged 70 or over, not coded as being prescribed anticoagulation, on the practice palliative care register or resident in a nursing home). A random sample of 275 such people from each practice were sent an information sheet and a consent form. They were selected from all eligible patients in each practice by a random number generator. People who returned the consent form were sent a screening leaflet inviting them to participate in screening for AF.

### Approaches to AF Screening

**Fig. 1:**
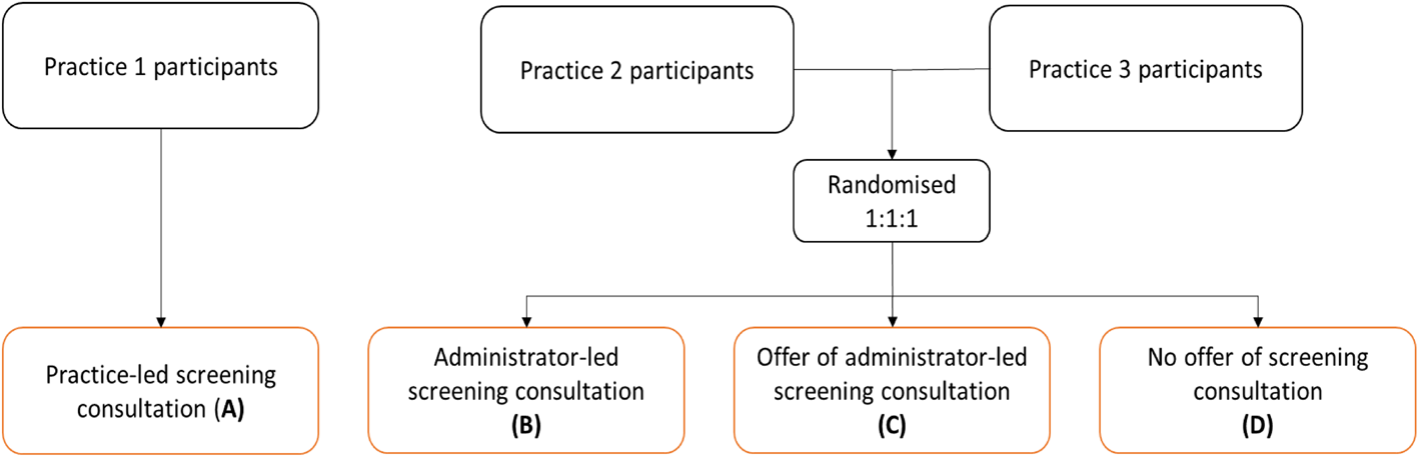
Summary of approaches to screening used All consultations were conducted remotely

In practice 1 (arm A of the study, see Fig. 1), the delivery and return of devices and remote support of patients were managed by practice staff. In practices 2 and 3, device delivery and return were managed by centralised study administrators. Participants who had provided consent were contacted by a member of the practice (practice 1) or by a study administrator (practices 2 and 3) to arrange delivery of the ECG device. Participants from practices 2 and 3 were provided (by random allocation) one of three levels of support for performing the ECGs: in Arm B: they were contacted by telephone to explain in detail how to perform the ECG; Arm C: they were offered by telephone (during the initial set-up call) a telephone screening consultation; Arm D: they were not offered a screening consultation, but told via telephone they could contact the study administrators should there be any queries. In practice 1 (Arm A), all patients received a screening consultation by a member of the practice staff. It was not practical to randomise participants to whether their support was managed by the practice or by a study administrator as during the COVID-19 pandemic it was important to keep practice involvement as simple as possible. The ECG device pack included simple instructions, a link to an internet instructional video, and a pre-paid envelope to return the device.

000000000000The study administrators monitored incoming ECG traces in arms B,C, and D and if the quality of recordings was consistently low according to a proprietary validated digital algorithm, [24, 25] the study administrators would call the participant and offer a screening consultation.

Participants were provided a hand-held single lead ECG device (Zenicor One, Zenicor Medical Systems AB) [26] by mail to generate a 30-s ECG trace, four times a day for three weeks, or if they experienced symptoms (for example, palpitations). Patients placed their thumbs or index fingers on the electrodes of the device. The device has been validated in other AF screening trials [11, 27].

The algorithm also flagged relevant ECGs as having possible AF, with these traces undergoing review and a final diagnosis of AF was made by a cardiologist. Results were sent to the participants’ general practices. Participants with AF or other significant arrhythmias were seen by a GP from their practice to discuss management including anticoagulation [18].

### Randomisation

All participants from practices 2 and 3 were randomised in a 1:1:1 ratio into arms B, C and D. Each consenting participant was randomly allocated through a random number generator at the study centre. Blinding was not possible.

### Outcomes

The primary outcome was the proportion of screened participants who provided at least 56 adequate-quality (i.e. without an algorithm ‘low quality’ tag) ECGs (two-thirds of a possible total of 84 ECGs from three weeks of screening).

Secondary outcomes were as follows: *screening uptake* (proportion of consented participants who provided at least one ECG trace), *screening performance* (proportion of ECG traces that were low quality; proportion of participants for whom > 50% of their ECG traces were adequate-quality; mean number of adequate-quality ECGs per participant), and *provision of extra support* (proportion of participants who received calls due to consistently low-quality ECGs).

### Sample size

The sample size calculations were based on the assumptions of 40% consent rate in the study, 0.05 significance level and 80% power. The anticipated proportion of adequate quality ECG traces per person (primary outcome) was 98% and thus a sample of 330 participants was adequate to detect a difference in the proportions of 1.2% or more. Analyses were performed in Stata v.18.

### Analysis

Outcomes were calculated for all participants in all arms as an overall assessment of feasibility. We report proportions, percentages, means, standard deviations (SDs) and 95% confidence intervals (CIs), as appropriate.

We compared arm A to arm B in order to investigate how, when a screening consultation is conducted, feasibility differed between practice staff-delivered and centralised administrator (SAFER staff) -delivered AF screening. For this purpose, we compared the proportions of arms A and B using the Z-test. Arms B, C, and D, which involved randomised participants, were compared using chi-square test for all outcomes measured as proportions in order to assess the intensity of support required for participants. Using the t-test and one-way analysis of variance (ANOVA) we compared the mean number of adequate-quality ECGs taken by participant across arms A versus B, and B versus C versus D respectively. All tests were performed at the 5% significance level.

### Patient and public involvement

We sought feedback from SAFER PPI members, [18] an external NHS PPI Group (Royal Papworth Hospital NHS Foundation Trust), and members of the public aged 70 years and over who were friends and family of the research team. They commented on the logistical aspects of remote delivery, and participant facing materials.

## Results

Practice characteristics are shown in Table 1. The three practices were in the least deprived five deciles of the country in terms of Index of Multiple Deprivation. Practice size varied between 7,000 and 12,000, and all practices had scores typical of the national performance on QOF (Quality and Outcomes Framework, which measures clinical and administrative performance outcomes that are financially incentivised). The median percentage of those with AF and CHADS2DS2-VASc ≥ 2 on anticoagulation for England was 90.6% [28].

**Table 1:** Characteristics of participating practices

Study practice number	Registered persons (to nearest 1000)	Rurality^a^	Deprivation decile^b^	QOF achievement out of 567	AF prevalence %	% of those with AF and CHADS_2_DS_2_-VASc ≥ 2 on anticoagulation
1	12,000	Urban	5 th	550.9	2.2	86.1
2	12,000	Rural	10 th	557	2.1	87.9
3	7000	Rural	7 th	567	4.5	93.9

*QOF* Quality and outcomes framework (measuring clinical and administrative performance outcomes that are financially incentivised), CHADS_2_DS_2_-VASc = scoring system with higher scores reflecting a higher risk of stroke (scores ≥ 2 should be offered anticoagulation unless contraindicated). Data comes from the Office for Health Improvement & Disparities’ Public Health Profiles [29] except where specified

^a^as judged by staff members from the practices

^b^1st decile is most deprived, 10th decile is least deprived according to Index of Multiple Deprivation (IMD)

**Fig. 2:**
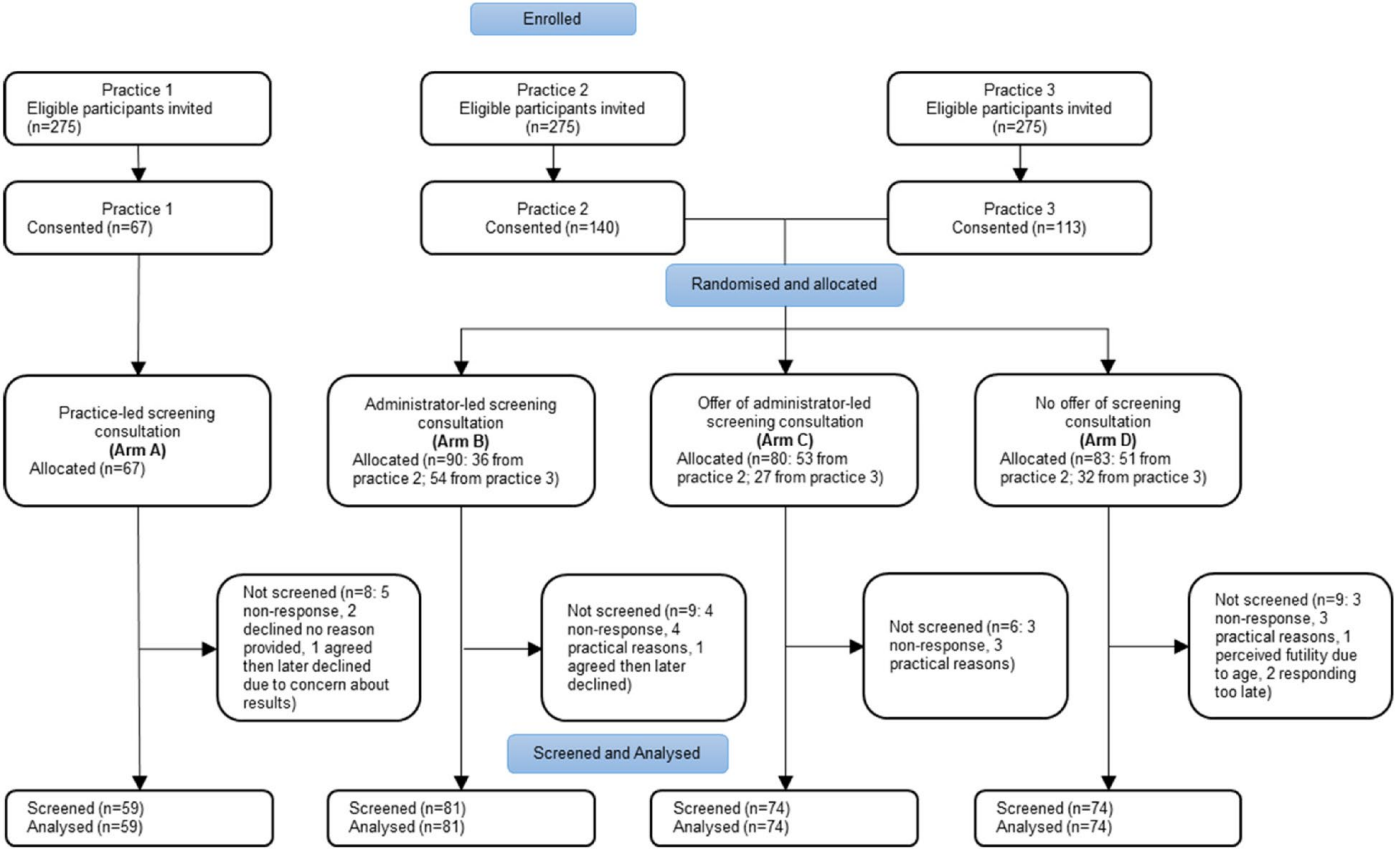
Participant flow All consultations were conducted remotely

Consent rates by practice varied from 24.3% to 50.9% (Fig. 2). Of the 32 participants who were allocated to an arm but then did not undertake screening, 15 did not respond to the invitation, 13 declined the invitation, two agreed to screening but later changed their mind, and two agreed to screening but responded too late. Reasons (summarised in Fig. 2) were generally for practical purposes, such as other commitments, or were not provided. All participants who were sent a device (*n* = 288) returned at least one ECG (59, 130 and 99 participants from practices 1, 2 and 3 respectively). As shown in Table 2, ages were similar across all arms.

**Table 2:** Age and gender of screened participants in each arm

	**Arm**			
	**Arm A (*n* = 59)**	**Arm B (*n* = 81)**	**Arm C (*n* = 74)**	**Arm D (*n* = 74)**
Age, mean (SD)	75.7 (5.6)	76.2 (5.0)	76.3 (5.1)	75.7 (4.5)
Gender, *n* (%)				
Female	27 (45.8)	40 (49.4)	39 (52.7)	35 (47.3)
Male	31 (52.5)	41 (50.6)	35 (47.3)	39 (52.7)
Unknown	1 (1.7)	0 (0.0)	0 (0.0)	0 (0.0)

*SD* Standard deviation

### Outcomes

**Fig. 3:**
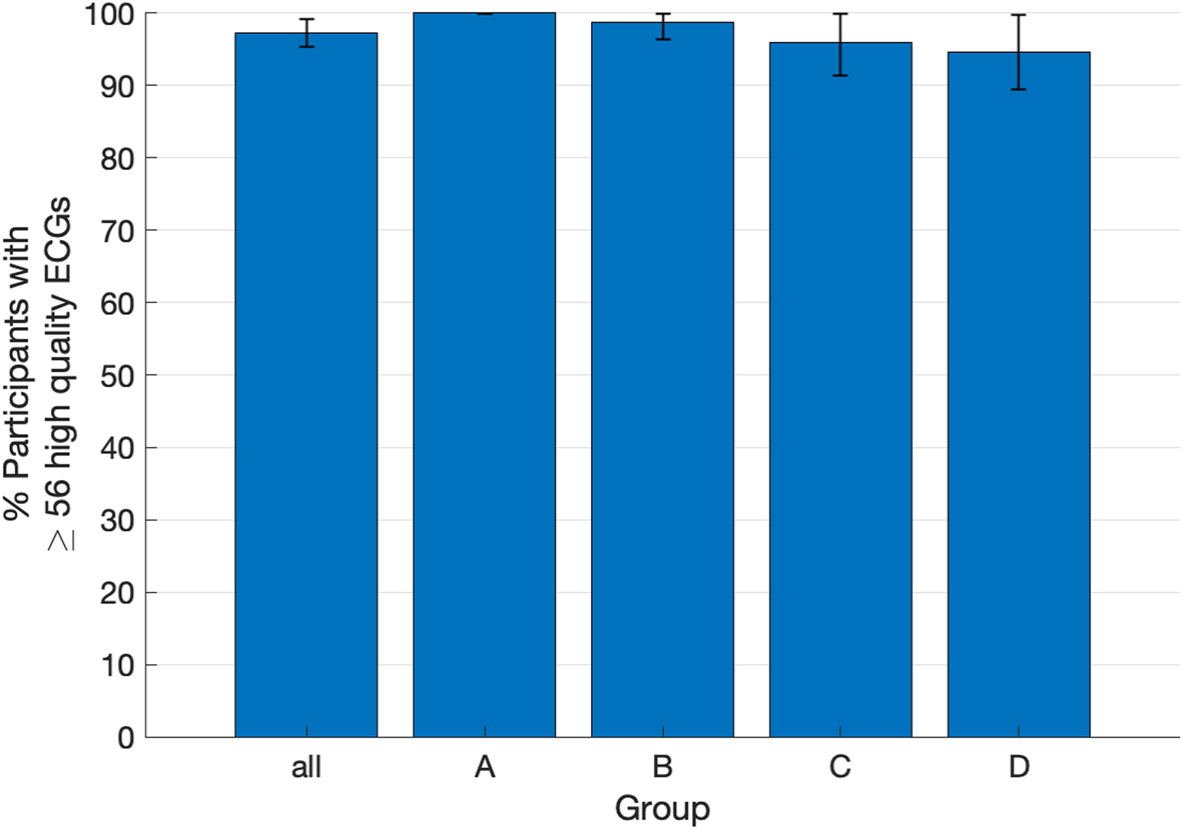
The primary outcome: The percentage of participants who recorded ≥ 56 adequate-quality ECGs The outcome for Arm A has no confidence interval as there was no variation around the proportion of participants with high-quality ECGs

97.2% (280/288) of screened participants recorded at least 56 adequate-quality traces. Performance by study arm is shown in Table 3 (see Additional file 1) and Fig. 3.

With respect to the primary outcome, in practice-led screening (arm A), 100.0% of participants recorded 56 adequate-quality ECG traces, compared to 98.8% in central administrator-led screening where all participants were given a screening consultation (arm B) (*p* = 0.39). In arms B (screening consultation given by default), C (screening consultation offered) and D (screening consultation not offered) 98.8%, 95.9%, and 94.6% of participants recorded this number of adequate-quality traces, respectively (Fig. 3). These were not significantly different (*p* = 0.35).

Overall 38.8% of invited participants consented. When separated by practice, consent rates were 24.4% for practice 1, 50.9% for practice 2, and 41.1% for practice 3 (*p* < 0.01).

Within administrator-led screening, the proportion of ECG traces that were low quality was associated with the level of support offered: 3.1% of ECGs were low quality in arm D (screening consultation not offered) compared to 2.4% in arm B (screening consultation given by default) and 2.2% in arm C (screening consultation offered) (*p* = 0.011).

There were significant differences between arms in the number of ECGs recorded by participants (*p* < 0.01). The mean number of ECGs per participant and the mean number of adequate-quality ECGs per participant, were both significantly higher in practice-led screening (arm A) than central administrator-led screening (arm B) (both *p* < 0.001) (see Table 2). These differences were small, with the differences in means spanning 95% CIs of 3.67—7.35 for the mean number of ECGs per participant and 3.38—7.81 for adequate quality ECGs respectively.

Only five out of 80 (6.25%) participants allocated to arm C opted for and received a screening consultation when offered. Overall, twelve participants were provided with a call for extra support due to consistently low quality ECGs, of which 10 received one call each, and 2 participants received two calls each. The mean duration of this call for ‘extra support’ was 7.6 min (SD 4.4). Only one participant, who was in Arm C, was determined to have ‘screening failure’ (no adequate-quality ECGs despite undertaking screening).

Overall, ten participants were diagnosed with AF and all 10 received anticoagulation by their GP [21].

## Discussion

### Summary

Over 97% of participants recorded at least 56 adequate-quality ECGs when carrying out self-screening for AF at home using a hand-held single-lead ECG. This proportion did not vary significantly whether supported by general practice, or by central administrators. Nor did it depend upon the amount of support received. The average number of adequate-quality ECGs and total number of ECGs was slightly higher in practice-led screening, and the proportion of low-quality ECGs was slightly higher if less support was offered, but neither of these differences are likely to be of clinical significance given the high number of adequate-quality ECGs provided in all arms.

### Strengths and limitations

This is the first study to explore whether AF screening can be carried out remotely without direct involvement of primary care. Although relatively small, it was of sufficient size to rule out clinically important differences in outcomes between the different delivery methods. The internal validity of our conclusion on the unimportance of intensity of support is strong, since this component was randomised. None of the administrators in the study had clinical training or prior experience of administering an AF screening programme, which might be similar to a real-world centrally-run programme.

The comparison of practice-led versus centrally-administered AF screening is weaker, since this component was non-randomised and only involved three practices. We cannot rule out that potential differences may have been masked by differences in practices or patients. Indeed, it is of note that the consent rate in the practice allocated to practice-led screening was half of that in the other practices.

The practices in this study may not be representative of UK practice. No practices were amongst the most deprived half of practices in England, so the results may not be applicable to more deprived areas. However, as a measure of the quality of AF care, the proportions of patients with a CHADS2DS2-VASc score of ≥ 2 on anticoagulation was not higher than the national average.

### Comparison with existing literature

Few studies have directly compared primary care staff to centralised administrators in delivering a screening programme, despite this comparison being crucial to minimise pressure on primary care. For those comparisons that have been done, in line with our findings, bowel cancer screening tests provided in primary care versus posted by central administrators, [30] and self-taken Human Papilloma Virus testing versus clinic-based tests, [31] are feasibly delivered by a centralised administration. However, there have been no such comparisons for more complicated technology, such as a hand-held ECG device that might need more clinical input. This study is therefore particularly important because of the risk of digitally-excluding elderly populations with such technology, and therefore it is important to test whether primary care staff or any clinically-trained staff are required [32]. We have shown that despite these concerns, people aged 70 and over who consented to screening can successfully use this technology with just written and video instructions, with delivery by non-clinical centralised administrators, and without the need to burden primary care.

The COVID-19 pandemic catalysed the creation and use of devices to remotely monitor health [13]. This added to the momentum towards the use of remote technology and patients measuring their own health parameters [33]. Remote devices also empower patients to take control of their health in the convenience of their own home [33]. We have shown that population screening for AF can be carried out at home following face-to-face training in general practice; [21] consumer-led detection of AF has already been demonstrated to be practicable using wearable devices [34, 35]. This study extends these findings to population based screening using hand-held devices without face-to-face contact. Thus, AF screening could now be considered as a remotely deliverable programme, alongside such examples as bowel cancer screening, [12] sexually transmitted infection screening, [16] cervical screening [15] and blood-pressure monitoring [13, 14].

Although 38.8% of invited participants consented to the study, there were significant differences in this between the practices. This might be explained by their different deciles of deprivation, with the most deprived (practice 1, 5 th most deprived decile), having the lowest consent rates in line with the evidence on the unrepresentativeness of research participation [36]. These figures are only slightly below the 51.3% consenting in STROKESTOP, a similar RCT screening trial that sent up to three invitations to participants as opposed to one in this study.

Thirty-two of the 320 participants who consented to the study did not undertake screening. While we do not have individual reasons for all of these participants, we published a qualitative interview study of the reasons for not screening for atrial fibrillation [32]. This suggests that practical reasons or an uncertainty as to whether screening would be beneficial might have been important in deciding not to screen. These accord with the data that we do have on reasons for non-participation [32].

### Implications for research and/or practice

The findings suggest that an AF screening programme could be delivered remotely and centrally, without the need to involve primary care or clinical staff in AF detection. It also suggests that remotely used hand-held ECG devices are successfully operated by elderly populations with only written and video instructions. It is possible that this might be relevant to the remote use of other digital interventions such as pedometers or respiratory devices [37, 38]. Policy makers might therefore have some confidence in being able to increase the detection of AF, in line with NHS priorities, in this way. However, demonstration that AF screening can be carried out in this way does not mean that such screening should be performed. Two underpowered trials of AF screening did not demonstrate a significant effect on stroke reduction. [19, 20] There are implications for primary care in terms of acting on ECG abnormalities identified, and there is a lack of evidence that screening in this way will reduce stroke risk [2, 39]. The SAFER trial is powered to answer these questions [40].

## Conclusions

A remotely delivered AF screening programme can be led by primary care staff or centrally-based administrators providing only written and video instructions. This has implications for meeting national health targets on detecting AF whilst minimising pressure on primary care and reducing the use of resources. Further implementation of enhanced AF detection should await stronger evidence that this activity does reduce risk of stroke.

## Supplementary Information

Additional file 1: Table 3. Screening uptake and performance by study arm.

## Data Availability

The datasets generated and/or analysed during the current study are not publicly available due to the SAFER trial still being in progress but are available from Andrew Dymond (SAFER@medschl.cam.ac.uk) on reasonable request.

## Abbreviations

AF: Atrial fibrillation
ECG: Electrocardiogram
NHS: UK National Health Service
SAFER: Screening for Atrial Fibrillation with ECG to Reduce Stroke trial
COVID-19: Coronavirus disease-2019
NIHR: National Institute for Health and Care Research
CRN: Clinical Research Network
GP: General practitioner
SD: Standard deviation
CI: Confidence interval
PPI: Patient and public involvement
QOF: Quality and outcomes framework
CHADS2DS2-VASc: Congestive heart failure, hypertension, age, diabetes mellitus, prior stroke or TIA or thromboembSolism, vascular disease, age, sex category scoring system for stroke risk in atrial fibrillation
STROKESTOP: Systematic ECG Screening for Atrial Fibrillation Among 75-Year-Old Subjects in the Region of Stockholm and Halland, Sweden trial
