# Supplementary material for: Screening for atrial fibrillation with or without general practice involvement: a controlled study
Source: BMC Prim Care. 2025 May 26;26:185. doi: 10.1186/s12875-025-02878-y (PMC12105410; doi:10.1186/s12875-025-02878-y)
Supplement: Supplementary file 1 — Additional file 1: Table 3. Screening uptake and performance by study arm. [file 12875_2025_2878_MOESM1_ESM.docx]

## Additional file 1. Table 3. Screening uptake and performance by study arm

| **Outcome** | Definition | Whole sample | Arm A | Arm B | Arm C | Arm D | A vs B | B vs C vs D |
| --- | --- | --- | --- | --- | --- | --- | --- | --- |
| Screening uptake | % of participants consenting to the study who provided at least one ECG trace | 90.0% (288/320)(CI: 86.7-93.3%) | 88.1% (59/67) | 90.0% (81/90) | 92.5% (74/80) | 89.2% (74/83) | p=0.704 | p=0.751 |
| Screening performance | Primary outcome: % of participants who recorded at least 56 adequate-quality ECGs | 97.2% (280/288) **(CI: 95.3-99.1%)** | 100.0%(59/59) **(CI: n/a)** | 98.8%(80/81) **(CI: 96.4-100.0%)** | 95.9%(71/74) **(CI: 91.5-100.0%)** | 94.6%(70/74) **(CI: 89.4-99.7%)** | p=0.401 | p=0.350 |
|  | % of ECG traces that were low quality ECGs | 2.5% (580/23,259) (CI: 2.3-2.7%) | 2.2% (110/5,061)(CI: 1.8-2.6%) | 2.4% (158/6,502)(CI: 2.1-2.8%) | 2.2% (131/5,824)(CI: 1.9-2.6%) | 3.1% (181/5,872)(CI: 2.6-3.5%) | p=0.477 | p=0.011 |
|  | % of participants that had >=50% adequate-quality ECGs | 99.7% (287/288)(CI: 99.0-100.0%) | 100.0% (59/59)(CI: n/a) | 100.0% (81/81)(CI: n/a) | 98.6% (73/74)(CI: 96.0-100.0%) | 100.0% (74/74)(CI: n/a) | p=0.818 | p=0.646^†^ |
|  | % of participants who performed at least 56 ECGs | 98.3% (283/288)(CI: 96.8-99.8%) | 100.0% (59/59)(CI: n/a) | 98.8% (80/81)(CI: 96.4-100.0%) | 97.3% (72/74)(CI: 93.6-100.0%) | 97.3% (72/74)(CI: 93.6-100.0%) | p=0.401 | p=0.528 |
|  | Mean number of ECGs taken by participants (SD) | 80.8 (9.0) | 85.8 (4.3) | 80.3 (6.1) | 78.7 (11.7) | 79.4 (10.1) | p<0.001 | p=0.576 |
|  | Mean number of adequate-quality ECGs taken by participants (SD) | 78.7 (10.0) | 83.9 (5.9) | 78.3 (7.0) | 76.9 (12.7) | 76.9 (11.2) | p<0.001 | p=0.628 |
| Provision of extra support | % of participants who received low quality calls | 4.2% (12/288)(CI: 1.9-6.5%)^‡^ | n/a | 4.9% (4/81)(CI: 0.2-9.7%) | 5.4% (4/74)(CI: 0.3-10.6%) | 5.4% (4/74)(CI: 0.3-10.6%) | p=0.085 | p=0.989 |

## Arm A: practice-led screening with support by practice-staff delivered screening consultations; Arm B: central administrator-led screening with support by an administrator delivered screening consultation; Arm C: central administrator-led screening with offer of an administrator delivered screening consultation; Arm D: central administrator-led screening with no offer of a screening consultation

## %: percentage; CI: 95% Confidence Interval

**^†^**Due to small frequencies, the Fisher exact test was used instead of Chi-square.

**^‡^**17 patients met the criteria. 5 did not receive a call due to logistic issues
